# Pregnancy Outcomes Based on Pre-Pregnancy Body Mass Index in Japanese Women

**DOI:** 10.1371/journal.pone.0157081

**Published:** 2016-06-09

**Authors:** Kimiko Enomoto, Shigeru Aoki, Rie Toma, Kana Fujiwara, Kentaro Sakamaki, Fumiki Hirahara

**Affiliations:** 1 Perinatal Center for Maternity and Neonate, Yokohama City University Medical Center, Yokohama, Kanagawa, Japan; 2 Department of Biostatistics and Epidemiology, Yokohama City University Graduate School of Medicine and University Medical Center, Yokohama, Kanagawa, Japan; 3 Department of Obstetrics and Gynecology, Yokohama City University Hospital, Yokohama, Kanagawa, Japan; Johns Hopkins Bloomberg School of Public Health, UNITED STATES

## Abstract

**Objective:**

To verify whether body mass index (BMI) classification proposed by the Institute of Medicine (IOM) is valid in Japanese women.

**Method:**

A study was conducted in 97,157 women with singleton pregnancies registered in the Japan Society of Obstetrics and Gynecology (JSOG) Successive Pregnancy Birth Registry System between January 2013 and December 2013, to examine pregnancy outcomes in four groups stratified by pre-pregnancy BMI category according to the 2009 criteria recommended by the Institute of Medicine (IOM). The groups comprised 17,724 underweight women with BMI <18.5, 69,126 normal weight women with BMI 18.5–24.9, 7,502 overweight women with BMI 25–29.9, and 2,805 obese women with BMI ≥30. The pregnancy outcomes were also compared among subgroups stratified by a gestational weight gain below, within, and above the optimal weight gain.

**Results:**

The higher the pre-pregnancy BMI, the higher the incidences of pregnancy-induced hypertension, gestational diabetes mellitus, macrosomia, cesarean delivery, postpartum hemorrhage, and post-term birth, but the lower the incidence of small for gestational age (SGA). In all pre-pregnancy BMI category groups, excess gestational weight gain was associated with a higher frequency of large for gestational age and macrosomia; poor weight gain correlated with a higher frequency of SGA, preterm birth, preterm premature rupture of membranes, and spontaneous preterm birth; and optimal weight gain within the recommended range was associated with a better outcome.

**Conclusion:**

The BMI classification by the IOM was demonstrated to be valid in Japanese women.

## Introduction

In 2009, the Institute of Medicine (IOM) classified body weight based on body mass index (BMI) as underweight (BMI <18.5 kg/m^2^), normal (BMI = 18.5–24.9 kg/m^2^), overweight (BMI = 25.0–29.9 kg/m^2^), and obese (BMI ≧30 kg/m^2^), and then published recommended guidelines for gestational weight gain according to these BMI categories [1].

Because there are many underweight women in Japan, the obesity classification used in Japan partially differs from that developed by the IOM. According to the criteria developed by the Japan Society for the Study of Obesity, women with a BMI of ≧25 kg/m^2^ are classified as obese, and are not further classified into any subtype [2]. Moreover, regarding gestational weight gain, the recommendation issued by the Japanese Ministry of Health, Labour and Welfare suggests that obese pregnant women with a BMI of ≧25 kg/m^2^ should receive treatments individually tailored to their needs [3] [4].

We conducted a large-scale retrospective study using the database of the Japan Society of Obstetrics and Gynecology (JSOG) Successive Pregnancy Birth Registry System to verify the validity of the IOM-BMI classification in Japanese women.

## Methods

**Fig 1:**
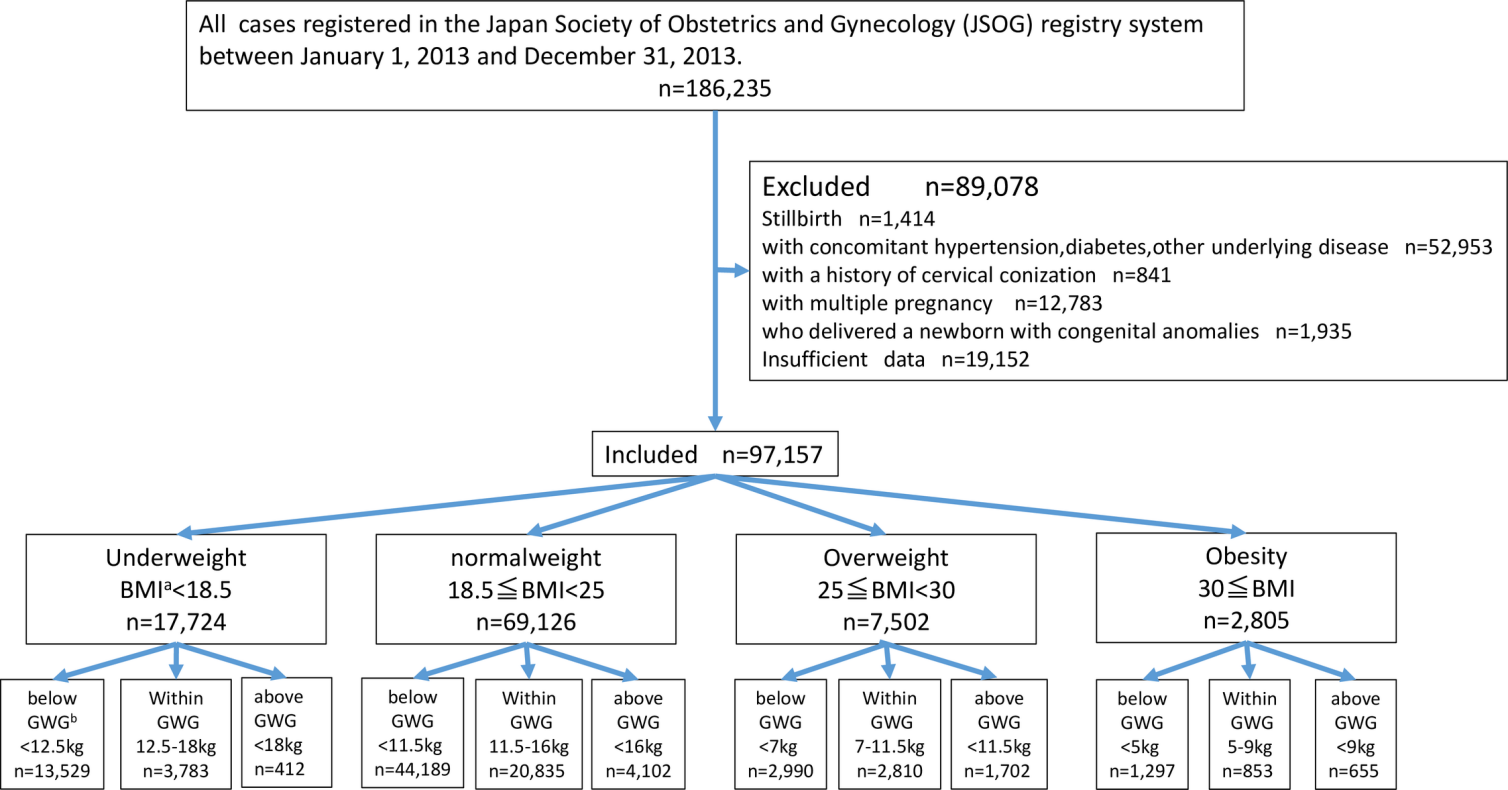
Flow diagram of study inclusion. ^a^BMI, body mass index; ^b^GWG, gestational weight gain.

This study was conducted after receiving approval by the ethics committee of the Yokohama City University Medical Center. The patient records was anonymized and de-identified prior to analysis. The present study was a retrospective investigation of women with singleton pregnancies who were included in the Japan Society of Obstetrics and Gynecology (JSOG) registry system. Approximately 280 secondary and tertiary hospitals participated in the JSOG Successive Pregnancy Birth Registry System, which collected information on successive deliveries occurring at gestational week 22 or later. A total of 186,235 women were registered in the system between January 1, 2013 and December 31, 2013. Women with concomitant hypertension or diabetes as the underlying disease, with a history of cervical conization, who delivered a newborn with congenital anomalies, or whose data were unknown, were excluded. After the exclusion, 97,157 women were included in the study (Fig 1).

Based on the 2009 IOM guidelines, 97,157 women were classified into the pre-pregnancy obese group (BMI ≥30 kg/m^2^), the pre-pregnancy overweight group (BMI = 25–29.9 kg/m^2^), the pre-pregnancy normal weight group (BMI = 18.5–25 kg/m^2^), or the pre-pregnancy underweight group (BMI <18.5 kg/m^2^).

The maternal characteristics recorded were age, primiparous rate, height, pre-pregnancy BMI, body weight at delivery, BMI at delivery, total gestational weight gain, and gestational age at delivery. The main outcomes were pregnancy induced hypertension (PIH), gestational diabetes mellitus (GDM), macrosomia, small for gestational age (SGA), large for gestational age (LGA), preterm birth, spontaneous preterm birth, preterm premature rupture of membranes (PPROM), induced preterm birth, cesarean delivery, severe postpartum hemorrhage, and post-term birth; the incidence of these outcomes was compared among the groups and analyzed. PIH was defined as a case in which hypertension (systolic blood pressure ≥140 mmHg and/or diastolic blood pressure ≥90 mmHg) developed after 20 weeks of gestation. GDM (by 75-g oral glucose tolerance test) was diagnosed when at least one of the following was found: fasting blood glucose level of ≥92 mg/dL, blood glucose level at 1 h of ≥180 mg/dL, blood glucose level at 2 h ≥153 mg/dL. Macrosomia was defined as a neonatal birthweight 4,000 g or more. SGA was defined as a neonatal birthweight below the 10th percentile of the reference curves of birthweight for gestational week. LGA was defined as a neonatal birth weight above the 90th percentile of the reference curves of birth weight for gestational week. Induced preterm birth was defined as preterm delivery by cesarean section or induction of labor due to maternal complications, such as preeclampsia or non-reassuring fetal status. Spontaneous preterm birth was defined as other than preterm birth medically indicated by cesarean section or labor induction. Postpartum hemorrhage with vaginal delivery (PPH with VD) was defined as an estimated blood loss of 500 mL or more; postpartum hemorrhage with cesarean delivery (PPH with C/S) was defined as an estimated blood loss of 1,000 mL or more. Post-term birth was defined as a delivery at ≥42 and 0/7ths weeks of gestation. Gestational age was determined based on the last menstrual period. If gestational age according to the last menstrual period differed by more than 7 days from that based on ultrasonography at less than 11 weeks, the latter was used to assign a gestational age [5].

Next, in order to investigate the relationship between weight gain during pregnancy and the pregnancy-delivery outcome, a stratified analysis was performed in three subgroups of gestational weight gain below, within, and above the optimal range, based on the IOM-recommended 2009 guidelines, to compare the frequency of PIH, GDM, SGA, high-fat diet, preterm birth, spontaneous preterm birth, PPROM, induced preterm birth, PPH with VD, PPH with C/S, macrosomia, and post-term birth in each group by pre-pregnancy BMI categories of underweight, normal weight, overweight, and obese.

Data were expressed as means ± standard deviation or frequencies (percentages). SPSS Statistics software version 23 (IBM Corp., Armonk, NY, USA) was used for the statistical analyses. One way analysis of variance (ANOVA) for continuous and a chi-square test for categorical data were used to compare the maternal characteristics and pregnancy outcomes among the four groups, with Bonferroni correction for multiple comparisons. A p value of <0.05 was considered statistically significant. Logistic regression analysis was used to estimate odds ratios (ORs) and 95% confidence intervals (CIs), adjusting for confounding variables, including maternal age, maternal height and parity. Generalized estimating equations (GEE) for logistic regression was used to adjust for clustering of deliveries by hospitals.

## Results

Of 97,157 pregnancies, the pre-pregnancy underweight group accounted for 18.2% (n = 17,724), the pre-pregnancy normal weight group for 71.1% (n = 69,126), the pre-pregnancy overweight group for 7.7% (n = 7,502), and the pre-pregnancy obese group for 2.9% (n = 2,805). Table 1 shows maternal characteristics according to the BMI categories. The primiparous rate was significantly higher in underweight and normal women (53.39%, 49.80%, 41.62%, and 44.14% in underweight, normal weight, overweight, and obese groups, respectively; p < 0.001). The gestational weight gain was significantly lower with increasing pre-pregnancy BMI value (10.27 ± 3.68, 10.11 ± 3.96, 7.98 ± 4.95, and 5.5 ± 5.57 kg in underweight, normal weight, overweight, and obese groups, respectively; p < 0.001).

**Table 1:** Comparison of maternal characteristics between pre-pregnancy underweight, normal weight, overweight and obese women.

	Underweight	Normal weight	Overweight	Obese	*p*-value*
	BMI^a^ < 18.5kg/m^2^	18.5≦BMI < 25kg/m^2^	25≦BMI < 30kg/m^2^	BMI≧30kg/m^2^	
	n = 17724	n = 69126	n = 7502	n = 2805	
maternal age(SD)	30.88(5.40)	31.95(5.37)	32.46(5.51)	32.04(5.32)	<0.001
primiparous rate(%)	9533(53.79%)	34424(49.8%)	3122(41.62%)	1238(44.14%)	<0.001
maternal body height (SD)	158.61(5.46)	158.16(5.42)	157.82(5.62)	157.98(5.77)	<0.001
pre-pregnancy BMI(SD)	17.59(0.75)	20.9(1.63)	26.93(1.39)	33.65(3.4)	<0.001
BMI at delivery (SD)	21.68(1.65)	24.95(2.21)	30.14(2.26)	35.86(3.78)	<0.001
gestational weight gain (SD)	10.27(3.68)	10.11(3.96)	7.98(4.95)	5.5(5.57)	<0.001
gestational week at delivery (SD)	38.23(2.26)	38.38(2.20)	38.2(2.44)	38.17(2.55)	<0.001

^a^BMI, body mass index.

Data are mean ± standard deviation or n (%), unless otherwise specified

* *P*-values represent the overall differences among the four groups that were evaluated using the ANOVA or a chi-square test.

Table 2 summarizes the incidences of PIH, GDM, SGA, LGA, preterm birth, spontaneous preterm birth, PPROM, induced preterm birth, cesarean delivery, PPH with VD, PPH with C/S, macrosomia, and post-term birth by BMI category. As the pre-pregnancy BMI increased, the incidence significantly increased for PIH, GDM, LGA, cesarean delivery, PPH with VD, PPH with C/S, macrosomia, and post-term birth (p < 0.001). On the other hand, the incidence of SGA significantly decreased with increasing pre-pregnancy BMI (p < 0.001). In addition, the lowest incidence was found in the normal weight group for preterm birth, PPROM, spontaneous preterm birth, and induced preterm birth.

**Table 2:** Comparison of pregnancy outcomes between pre-pregnancy underweight, normal weight, overweight and obese women.

		Underweight	Normal weight	Overweight	Obesity	*p*-value*
		BMI^a^ < 18.5kg/m^2^	18.5≦BMI < 25kg/m^2^	25≦BMI < 30kg/m^2^	BMI≧30kg/m^2^	
		n = 17724	n = 69126	n = 7502	n = 2805	
PIH^b^						
	%(n)	3.34%(n = 592)	4.68%(n = 3237)	10.08% (n = 756)	14.65%(n = 411)	<0.001
	OR(95%CI)	0.724(0.657–0.798)	1	2.373 (2.125–2.649)	3.693 (3.170–4.302)	
GDM^c^						
	%(n)	2.66%(n = 472)	3.86%(n = 2665)	10.72% (n = 804)	20.61%(n = 578)	<0.001
	OR(95%CI)	0.737(0.663–0.819)	1	2.905 (2.645–3.189)	6.582 (5.833–7.427)	
SGA^d^						
	%(n)	13.21%(n = 2341)	8.67%(n = 5992)	7.24% (n = 543)	7.06% (n = 198)	<0.001
	OR(95%CI)	1.657 (1.557–1.764)	1	0.807 (0.731–0.891)	0.790(0.688–0.908)	
LGA^e^						
	%(n)	5.46%(n = 967)	10.05%(n = 6944)	17.36% (n = 1302)	22.60% (n = 634)	<0.001
	OR(95%CI)	0.50 (0.466–0.536)	1	1.96 (1.834–2.094)	2.709 (2.457–2.988)	
Preterm birth						
	%(n)	12.2%(n = 2154)	10.2%(n = 7063)	11.6%(n = 873)	12.1% (n = 338)	<0.001
	OR(95%CI)	1.236(1.164–1.313)	1	1.133 (1.043–1.230)	1.187 (1.044–1.350)	
Spontaneous preterm birth						
	%(n)	10.2%(n = 1810)	8.3%(n = 5751)	8.8%(n = 662)	9.1% (n = 256)	<0.001
	OR(95%CI)	1.275(1.196–1.359)	1	1.037 (0.937–1.134)	1.085 (0.937–1.256)	
PPROM^f^						
	%(n)	3.78%(n = 670)	3.01%(n = 2081)	3.35%(n = 251)	3.35% (n = 94)	<0.001
	OR(95%CI)	1.261(1.145–1.3890)	1	1.114 (0.976-Ⅰ.273)	1.115 (0.893–1.392)	
Induce preterm birth						
	%(n)	1.9%(n = 344)	1.9%(n = 1317)	2.8%(n = 211)	2.9% (n = 82)	<0.001
	OR(95%CI)	1.034(0.922–1.160)	1	1.509 (1.302–1.748)	1.573 (1.232–2.010)	
Total C/S^g^						
	%(n)	22.54%(n = 3995)	26.95%(n = 18627)	36.78%(n = 2759)	42.96% (n = 1205)	<0.001
	OR(95%CI)	0.857 (0.818–0.897)	1	1.506 (1.420–1.597)	2.042 (1.855–2.248)	
PPH^h^ with VD^i^						
	%(n)	19.57%(n = 3468)	20.94% (n = 14476)	21.22% (n = 1592)	22.07% (n = 619)	<0.001
	OR (95%CI)	0.886 (0.842–0.932)	1	1.070 (0.999–1.146)	1.104 (0.995–1.226)	
PPH with C/S						
	% (n)	4.74% (n = 840)	7.64% (n = 5281)	11.37% (n = 853)	13.80% (n = 387)	<0.001
	OR (95%CI)	0.659 (0.601–0.722)	1	1.461 (1.338–1.595)	1.91 (1.684–2.167)	
macrosomia>4000g						
	% (n)	0.27% (n = 47)	0.69% (n = 474)	1.76% (n = 132)	3.07% (n = 86)	<0.001
	OR (95%CI)	0.375 (0.280–0.503)	1	2.609 (2.159–3.154)	4.599 (3.667–5.767)	
post-term pregnancy						
	% (n)	0.16% (n = 28)	0.24% (n = 169)	0.41% (n = 31)	0.57% (n = 16)	<0.001
	OR^j^ (95%CI^k^)	0.623 (0.431–0.901)	1	1.879 (1.250–2.824)	2.524 (1.546–4.120)	

^a^BMI, body mass index

^b^PIH, pregnancy-induced hypertension

^c^GDM, gestational diabetes mellitus

^d^SGA, small for gestational age

^e^LGA, large for gestational age

^f^PPROM, preterm prelabor rupture of membranes

^g^C/S,cesarian section

^h^PPH,postpartum hemorrhage

^i^VD,Vaginal delivery

^j^OR, odds ratio

^k^CI, confidence interval

Logistic regression was used to adjust for confounding variables, including maternal age, maternal height and parity, and generalized estimating equations (GEE) for logistic regression was used to adjust for the clustering of deliveries by hospitals. The results were expressed as odds ratios (OR) and 95% confidence intervals (CI).

Tables 3 through 6 summarize the incidences of pregnancy outcomes in subgroups stratified by gestational weight gain below, within, and above the IOM-recommended range for PIH, SGA, LGA, preterm birth, PPROM, spontaneous preterm birth, induced preterm birth, cesarean delivery, PPH with VD, PPH with C/S, macrosomia, and post-term birth.

**Table 3:** Pregnancy outcomes for underweight women in reference to weight gain.

		below	within	above	
underweight		GWG^a^ * *<12.5kg	GWG: 12.5-18kg	GWG>18kg	*p*-value*
		N = 13529	N = 3783	N = 412	
PIH^b^					
	% (n)	3.0% (n = 407)	4.0% (n = 150)	8.5% (n = 35)	<0.001
	OR (95%CI)	0.726 (0.594–0.887)	1	2.380 (1.635–3.464)	
GDM^c^					
	% (n)	3.0% (n = 405)	1.6% (n = 61)	1.5% (n = 6)	<0.001
	OR (95%CI)	1.703 (1.275–2.274)	1	1.030 (0.476–2.229)	
SGA^d^					
	% (n)	15% (n = 2032)		5.6% (n = 23)	<0.001
	OR (95%CI)	2.142 (1.849–2.482)	7.6% (n = 286)	0.774 (0.516–1.161)	
LGA^e^			1		
	% (n)	3.8% (n = 518)	10.3% (n = 388)	14.8% (n = 61)	<0.001
	OR (95%CI)	0.352 (0.310–0.399)	1	1.443 (1.067–1.950)	
Preterm birth					
	% (n)	14.6% (n = 1979)	4.4% (n = 167)	1.9% (n = 8)	<0.001
	OR (95%CI)	3.857 (3.227–4.609)	1	0.418 (0.202–0.869)	
Spontaneous preterm birth					
	% (n)	12.2% (n = 1651)	3.1% (n = 116)	0.7% (n = 3)	<0.001
	OR (95%CI)	4.710 (3.805–5.830)	1	0.226 (0.071–0.716)	
PPROM^f^					
	% (n)	4.5% (n = 614)	1.4% (n = 54)	0.5% (n = 2)	<0.001
	OR (95%CI)	3.431 (2.526–4.661)	1	0.319 (0.075–1.349)	
Induced preterm birth					
	% (n)	2.4% (n = 328)	1.4% (n = 51)	1.2% (n = 5)	0.003
	OR (95%CI)	1.619 (1.189–2.204)	1	0.895 (0.354–2.260)	
Total C/S^g^					
	% (n)	23.5% (n = 3174)	19.5% (n = 739)	19.9% (n = 82)	0.018
	OR (95%CI)	1.138 (1.037–1.247)	1	1.205 (0.915–1.587)	
PPH^h^ with VD^i^					
	% (n)	18.2% (n = 2461)	23.7% (n = 898)	26.5% (n = 109)	<0.001
	OR (95%CI)	0.736 (0.675–0.803)	1	1.081 (0.844–1.384)	
PPH with C/S					
	% (n)	4.7% (n = 635)	4.8% (n = 181)	5.8% (n = 24)	0.025
	OR (95%CI)	0.872 (0.728–1.043)	1	1.453 (0.904–2.336)	
macrosomia>4000g					
	% (n)	0.1% (n = 16)	0.5% (n = 20)	2.7% (n = 11)	<0.001
	OR (95%CI)	0.215 (0.118–0.392)	1	5.292 (2.466–11.355)	
post-term pregnancy					
	% (n)	0.16% (n = 21)	0.16% (n = 6)	0.24% (n = 1)	0.925
	OR^j^ (95%CI^k^)	0.998 (0.417–2.389)	1	1.50 (0.186–12.113)	

^a^GWG, gestational weight gain, body mass index

^b^PIH, pregnancy-induced hypertension

^c^ GDM, gestational diabetes mellitus

^d^SGA, small for gestational age

^e^ LGA, large for gestational age

^f^PPROM, preterm prelabor rupture of membranes

^g^C/S,cesarian section

^h^PPH,postpartum hemorrhage

^i^VD,Vaginal delivery

^j^OR, odds ratio

^k^CI, confidence interval

* Logistic regression was used to adjust for confounding variables, including maternal age, maternal height and parity, and generalized estimating equations (GEE) for logistic regression was used to adjust for the clustering of deliveries by hospitals. The results were expressed as odds ratios (OR) and 95% confidence intervals (CI).

* * IOM Recommendation for gestational weight gain: underweight, 12.5–18 kg; normal, 11.5–16 kg; overweight, 7–11.5 kg; and obese, 5–9 kg.

**Table 4:** Pregnancy outcomes for normal weight women in reference to weight gain.

		below	within	above	
normalweight		GWG^a^ * *<11.5kg	GWG: 11.5-16kg	GWG>16kg	*p*-value*
		n = 44189	n = 20835	n = 4102	
PIH^b^					
	% (n)	4.4% (n = 1938)	4.7% (n = 976)	7.9% (n = 323)	<0.001
	OR (95%CI)	0.904 (0.833–0.980)	1	1.852 (1.589–2.157)	
GDM^c^					
	% (n)	4.6% (n = 2031)	2.4% (n = 504)	3.2% (n = 130)	<0.001
	OR (95%CI)	1.814 (1.633–1.770)	1	1.477 (1.233–1.770)	
SGA^d^					
	% (n)	10.4% (n = 4575)	6.0% (n = 1254)	4.0% (n = 163)	<0.001
	OR (95%CI)	1.764 (1.647–1.889)	1	0.659 (0.554–0.784)	
LGA^e^					
	% (n)	7.5% (n = 3322)	13.2% (n = 2754)	21.2% (n = 868)	<0.001
	OR (95%CI)	0.544 (0.517–0.572)	1	1.734 (1.592–1.888)	
Preterm birth					
	% (n)	13.3% (n = 5891)	4.8% (n = 994)	4.3% (n = 178)	<0.001
	OR (95%CI)	3.042 (2.851–3.246)	1	0.916 (0.774–1.084)	
Spontaneous preterm birth					
	% (n)	11.1% (n = 4903)	3.5% (n = 733)	2.7% (n = 110)	<0.001
	OR (95%CI)	3.386 (3.156–3.633)	1	0.763 (0.623–0.934)	
PPROM^f^					
	% (n)	3.9% (n = 1741)	1.4% (n = 292)	1.2% (n = 48)	<0.001
	OR (95%CI)	2.932 (2.570–3.334)	1	0.810 (0.601–1.091)	
Induced preterm birth					
	% (n)	2.2% (n = 988)	1.3% (n = 261)	1.7% (n = 68)	<0.001
	OR (95%CI)	1.793 (1.549–2.075)	1	1.342 (1.035–1.740)	
Total C/S^g^					
	% (n)	28.2% (n = 12446)	24.3% (n = 5062)	27.3% (n = 1119)	<0.001
	OR (95%CI)	1.117(1.071–1.166)	1	1.358 (1.252–1.474)	
PPH^h^ with VD^i^					
	% (n)	19.2%(n = 8496)	23.5% (n = 4893)	26.5% (n = 1087)	<0.001
	OR (95%CI)	0.806 (0.772–0.840)	1	1.110 (1.026–1.200)	
PPH with C/S					
	% (n)	7.7% (n = 3389)	7.3% (n = 1531)	8.8% (n = 361)	<0.001
	OR (95%CI)	0.949 (0.888–1.104)	1	1.420 (1.251–1.611)	
macrosomia>4000					
	% (n)	0.3% (n = 149)	1.0% (n = 214)	2.7%(n = 111)	<0.001
	OR (95%CI)	0.329 (0.267–0.406)	1	2.693(2.163–3.353)	
post-term pregnancy					
	% (n)	0.2%(n = 86)	0.3% (n = 58)	0.6%(n = 25)	<0.001
	OR^j^ (95%CI^k^)	0.726 (0.528–0.997)	1	2.096 (1.315–3.343)	

^a^GWG, gestational weight gain, body mass index

^b^PIH, pregnancy-induced hypertension

^c^ GDM, gestational diabetes mellitus

^d^SGA, small for gestational age

^e^ LGA, large for gestational age

^f^PPROM, preterm prelabor rupture of membranes

^g^C/S,cesarian section

^h^PPH,postpartum hemorrhage

^i^VD,Vaginal delivery

^j^OR, odds ratio

^k^CI, confidence interval

* Logistic regression was used to adjust for confounding variables, including maternal age, maternal height and parity, and generalized estimating equations (GEE) for logistic regression was used to adjust for the clustering of deliveries by hospitals. The results were expressed as odds ratios (OR) and 95% confidence intervals (CI).

* * IOM Recommendation for gestational weight gain: underweight, 12.5–18 kg; normal, 11.5–16 kg; overweight, 7–11.5 kg; and obese, 5–9 kg.

**Table 5:** Pregnancy outcomes for overweight women in reference to weight gain.

		below	within	above	
overweight		GWG^a^ * *<7kg	GWG: 7–11.5kg	GWG>11.5kg	*p*-value*
		n = 2990	n = 2810	n = 1702	
PIH^b^					
	% (n)	9.1% (n = 273)	9.3% (n = 261)	13.0% (n = 222)	<0.001
	OR (95%CI)	0.972 (0.821–1.150)	1	1.542 (1.277–1.864)	
GDM^c^					
	% (n)	15.0% (n = 447)	9.0% (n = 252)	6.2% (n = 105)	<0.001
	OR (95%CI)	1.749 (1.472–2.078)	1	0.711 (0.561–0.901)	
SGA^d^					
	% (n)	9.2% (n = 275)	6.4% (n = 179)	5.2% (n = 89)	<0.001
	OR (95%CI)	1.489 (1.212–1.829)	1	0.863 (0.663–1.123)	
LGA^e^					
	% (n)	12.1% (n = 363)	17.4% (n = 489)	26.4% (n = 450)	<0.001
	OR (95%CI)	0.653 (0.560–0.761)	1	1.655 (1.433–1.912)	
Preterm birth					
	% (n)	17.0% (n = 508)	8.5% (n = 240)	7.3% (n = 125)	<0.001
	OR (95%CI)	2.178 (1.830–2.593)	1	0.876 (0.690–1.112)	
Spontaneous preterm birth					
	% (n)	13.8% (n = 413)	6.2% (n = 173)	4.5% (n = 76)	<0.001
	OR (95%CI)	2.429 (1.980–2.978)	1	0.732 (0.550–0.974)	
PPROM^f^					
	% (n)	4.9% (n = 146)	2.3% (n = 66)	2.3% (n = 39)	<0.001
	OR (95%CI)	2.153 (1.574–2.943)	1	0.989 (0.665–1.471)	
Induced preterm birth					
	% (n)	3.2% (n = 95)	2.4% (n = 67)	2.9% (n = 49)	0.219
	OR (95%CI)	1.334 (0.961–1.851)	1	1.261 (0.840–1.895)	
Total C/S^g^					
	% (n)	38.5% (n = 1151)	35.3% (n = 991)	36.3% (n = 617)	0.029
	OR (95%CI)	1.101 (0.987–1.229)	1	1.176 (1.041–1.328)	
PPH^h^ with VD^i^					
	% (n)	19.4% (n = 580)	21.4% (n = 600)	24.2% (n = 412)	0.068
	OR (95%CI)	0.911 (0.800–1.037)	1	1.097 (0.945–1.002)	
PPH with C/S					
	% (n)	12.0% (n = 358)	11.0% (n = 308)	11.0% (n = 187)	0.464
	OR (95%CI)	1.046 (0.886–1.236)	1	1.133 (0.928–1.383)	
macrosomia>4000					
	% (n)	1.1% (n = 34)	1.2% (n = 35)	3.7% (n = 63)	<0.001
	OR (95%CI)	0.894 (0.564–1.416)	1	3.088 (1.955–4.875)	
post-term pregnancy					
	% (n)	0.2% (n = 7)	0.4% (n = 12)	0.7% (n = 12)	0.108
	OR^j^ (95%CI^k^)	0.582 (0.270–1.254)	1	1.527 (0.690–3.378)	

^a^GWG, gestational weight gain, body mass index

^b^PIH, pregnancy-induced hypertension

^c^ GDM, gestational diabetes mellitus

^d^SGA, small for gestational age

^e^ LGA, large for gestational age

^f^PPROM, preterm prelabor rupture of membranes

^g^C/S,cesarian section

^h^PPH,postpartum hemorrhage

^i^VD,Vaginal delivery

^j^OR, odds ratio

^k^CI, confidence interval

* Logistic regression was used to adjust for confounding variables, including maternal age, maternal height and parity, and generalized estimating equations (GEE) for logistic regression was used to adjust for the clustering of deliveries by hospitals. The results were expressed as odds ratios (OR) and 95% confidence intervals (CI).

* * IOM Recommendation for gestational weight gain: underweight, 12.5–18 kg; normal, 11.5–16 kg; overweight, 7–11.5 kg; and obese, 5–9 kg.

**Table 6:** Pregnancy outcomes for obese women in reference to weight gain.

		below	within	above	
obese		GWG^a^ * *<5kg	GWG: 5-9kg	GWG>9kg	*p*-value*
		n = 1297	n = 853	n = 655	
PIH^b^					
	% (n)	14.3% (n = 185)	14.3% (n = 122)	15.9% (n = 104)	0.492
	OR (95%CI)	0.982 (0.778–1.240)	1	1.147 (0.852–1.545)	
GDM^c^					
	% (n)	21.7% (n = 331)	16.4% (n = 140)	16.3% (n = 107)	<0.001
	OR (95%CI)	1.703 (1.323–2.193)	1	1.030 (0.765–1.386)	
SGA^d^					
	% (n)	7.4% (n = 112)	5.6% (n = 48)	5.8% (n = 38)	0.036
	OR (95%CI)	1.628 (1.085–2.444)	1	1.108 (0.714–1.720)	
LGA^e^					
	% (n)	14.0% (n = 213)	24.2% (n = 206)	32.8% (n = 215)	<0.001
	OR (95%CI)	0.595 (0.476–0.742)	1	1.470 (1.162–1.860)	
Preterm birth					
	% (n)	13.1% (n = 199)	11.1% (n = 95)	6.7% (n = 44)	<0.001
	OR (95%CI)	1.459 (1.134–1.877)	1	0.591 (0.421–0.828)	
Spontaneous preterm birth					
	% (n)	10.6% (n = 161)	8.3% (n = 71)	3.7% (n = 24)	<0.001
	OR (95%CI)	1.580 (1.201–2.078)	1	0.429 (0.279–0.659)	
PPROM^f^					
	% (n)	3.5% (n = 54)	3.4% (n = 29)	1.7% (n = 11)	0.008
	OR (95%CI)	1.280 (0.833–1.967)	1	0.486 (0.265–0.890)	
Induced preterm birth					
	% (n)	2.5% (n = 38)	2.8% (n = 24)	3.1% (n = 20)	0.923
	OR(95%CI)	1.039(0.617–1.751)	1	1.124 (0.630–2.004)	
Total C/S^g^					
	%(n)	35.6% (n = 542)	43.0% (n = 367)	45.2% (n = 296)	0.063
	OR(95%CI)	0.934 (0.790–1.104)	1	1.171 (0.937–1.462)	
PPH^h^ with VD^i^					
	%(n)	18.7% (n = 285)	21.7% (n = 185)	22.7% (n = 149)	0.907
	OR(95%CI)	1.038 (0.857–1.256)	1	0.994 (0.766–1.290)	
PPH with C/S					
	%(n)	12.1% (n = 184)	11.7% (n = 100)	15.7% (n = 103)	0.031
	OR(95%CI)	1.220 (0.942–1.581)	1	1.485 (1.106–1.995)	
macrosomia>4000					
	%(n)	1.4% (n = 21)	3.3% (n = 28)	5.6% (n = 37)	<0.001
	OR(95%CI)	0.462 (0.260–0.818)	1	1.592 (0.906–2.799)	
post-term pregnancy					
	%(n)	0.2% (n = 3)	1.17% (n = 10)	0.5% (n = 3)	0.035
	OR^j^(95%CI ^k^)	0.203 (0.056–0.736)	1	0.380(0.103–1.393)	

^a^GWG, gestational weight gain, body mass index

^b^PIH, pregnancy-induced hypertension

^c^GDM, gestational diabetes mellitus

^d^SGA, small for gestational age

^e^ LGA, large for gestational age

^f^PPROM, preterm prelabor rupture of membranes

^g^C/S,cesarian section

^h^PPH,postpartum hemorrhage

^i^VD,Vaginal delivery

^j^OR, odds ratio

^k^CI, confidence interval

* Logistic regression was used to adjust for confounding variables, including maternal age, maternal height and parity, and generalized estimating equations (GEE) for logistic regression was used to adjust for the clustering of deliveries by hospitals. The results were expressed as odds ratios (OR) and 95% confidence intervals (CI).

* * IOM Recommendation for gestational weight gain: underweight, 12.5–18 kg; normal, 11.5–16 kg; overweight, 7–11.5 kg; and obese, 5–9 kg.

In the underweight and normal weight groups, the incidences of PIH, LGA, and macrosomia were significantly higher with increasing gestational weight gain (p < 0.001), and the incidences of SGA, preterm birth, PPROM, spontaneous preterm birth, and induced preterm birth were significantly higher in the subgroup with gestational weight gain below the optimal range (p < 0.001). In both groups, the frequency of cesarean delivery was lowest in the subgroup within the recommended weight gain.

In the overweight group, the incidences of PIH and LGA were significantly higher with increasing gestational weight gain, and the incidence of macrosomia was significantly higher in the subgroup above the optimal weight gain (p < 0.001). In addition, the incidences of SGA, preterm birth, PPROM, and spontaneous preterm birth were significantly higher in the subgroup below the optimal weight gain (p < 0.001). The incidence of cesarean delivery was lowest in the subgroup within the recommended weight gain.

In the obese group, the incidences of LGA, and macrosomia were significantly higher with increasing gestational weight gain (p < 0.001), and the incidences of SGA, preterm birth, PPROM and spontaneous preterm birth were significantly higher in the subgroup with gestational weight gain below the optimal range (p < 0.05); the incidence of PIH and cesarean delivery were not different by weight gain during pregnancy.

## Discussion

The four pre-pregnancy BMI category groups demonstrated marked differences in the pregnancy outcome profile, indicating that the IOM-BMI classification is valid in Japanese women as well. In all groups, the gestational weight gain above the optimal range was associated with a higher incidence of LGA; the weight gain below the optimal range correlated with a higher incidence of SGA, preterm birth, PPROM, and spontaneous preterm birth; and the weight gain within the recommended range was associated with a better outcome. However, only the obese group did not show a difference in the incidence of PIH by gestational weight gain or a reduction in the incidence of cesarean delivery, even within the recommended weight gain.

The BMI classification by the IOM was found to be valid in Japanese women.

There were differences in the incidence of adverse pregnancy outcomes according to IOM recommended BMI categories. Due to a trend toward a higher proportion of leaner women and a lower proportion of obese individuals in the Japanese pregnant population, we defined those with BMI ≥25.0 as obese, in contrast to the IOM classification. However, the present study results indicated that Japanese pregnant women should also be categorized into an overweight group of pre-pregnant BMI 25–29.9 and an obese group with BMI ≥30. In addition, the group with BMI ≥30 did not show a difference in the incidence of PIH by weight control status during pregnancy or a reduction in cesarean delivery rate, even when the body weight was kept within the recommended range. These findings also indicated that weight reduction prior to pregnancy was important in improving pregnancy outcomes in obese women with BMI ≥30 in pregnancy. Poston et al. [6] reported findings of one of the largest randomised trials to assess the effects of interventions addressing diet and physical activity in obese pregnant women, demonstrating that a complex intervention in pregnant women with obesity is effective at improving diet quality and physical activity, reducing gestational weight gain, and decreasing surrogate measures of maternal body fatness, however the intervention does not prevent development of gestational diabetes nor change the incidence of LGA infants in this population, neither was evidence noted of a benefit on other pregnancy outcomes, including pre-eclampsia, which is associated with raised BMI. They recommend a shift in research focus towards renewed efforts towards effective public health measures that prevent obesity in women of reproductive age, which supports our suggestion.

The incidence rates of LGA and macrosomia were higher in women who had excess weight gain during pregnancy. Johnson et al. [7] reported a retrospective cohort study on gestational weight gain and pregnancy outcomes in 3,191 full-term women with singleton pregnancies, demonstrating that excess weight gain was associated with higher incidence of macrosomia and cesarean delivery. Other studies also reported similar findings [7–14], and our present study results are consistent with their conclusion.

The incidences of SGA, preterm birth, PPROM, and spontaneous preterm birth increased when the weight gain was below optimal. We previously reported that below optimal weight gain during pregnancy was associated with an increased risk of spontaneous preterm birth in underweight women [15]; however, the correlation between poor weight gain and spontaneous preterm birth is not limited to the underweight group, but is commonly seen in all groups. It is speculated that poor weight gain during pregnancy stimulates the production of stress hormones, such as epinephrine and cortisol, which in turn stimulate maternal corticosterone-releasing-hormone (CRH) secretion and prostaglandin production, making the body susceptible to uterine contractions; moreover, deficiency of nutrients such as iron and zinc impairs immunity and promotes chorioamnionitis, leading to preterm birth [16,17]. Stotland et al. [12] reported similar results, indicating the necessity of appropriate weight gain in both overweight and obesity.

The present study has limitations. First, our data set included only Japanese women, and it is unclear whether the results can be extrapolated to women of other ethnic groups. Second, we used the database of the JSOG Successive Pregnancy Birth Registry in tertiary centers. This may have introduced selective bias in the patient background characteristics. Third, we calculated the gestational weight gain as the change over the entire pregnancy period, and did not assess weekly weight gain.

In Japan, pre-pregnancy BMI is currently classified into three categories: BMI <18.5, 18.5 to <25.0, and ≥25.0. On the basis of the present study results, we propose that the Japanese category of BMI ≥25.0 should also be further divided into overweight (BMI 25.0–29.9) and obese (BMI ≥30), in keeping with the IOM-recommended BMI classification. Our results also demonstrated that obese women with BMI ≥30 had a higher risk of adverse pregnancy outcome, and that gestational weight control did not have an impact on the cesarean delivery rate, supporting the importance of pre-pregnancy weight control within the normal range.
